# The flavonoid content and antiproliferative, hypoglycaemic, anti-inflammatory and free radical scavenging activities of *Annona dioica* St. Hill

**DOI:** 10.1186/1472-6882-13-14

**Published:** 2013-01-11

**Authors:** Anelise S N Formagio, Candida A L Kassuya, Frederico Formagio Neto, Carla R F Volobuff, Edna K K Iriguchi, Maria do C Vieira, Mary Ann Foglio

**Affiliations:** 1 Faculdade de Ciências Agrárias, Universidade Federal da Grande Dourados, Dourados, MS, Brazil; 2 Faculdade de Ciências da Saúde, Universidade Federal da Grande Dourados, Dourados, MS, Brazil; 3 Faculdade de Ciências Biológicas e Ambiental, Universidade Federal da Grande Dourados, Dourados, MS, Brazil; 4 Centro Pluridisciplinar de Pesquisas Químicas, Biológicas e Agrícolas, Universidade de Campinas, Campinas, Brazil

**Keywords:** *Annona dioica*, Flavonoids, Antioxidant, Hypoglycaemic, Antiproliferative, Anti-inflammatory

## Abstract

**Background:**

*Annona dioica* St. Hill (Annonacaeae) is a Brazilian plant used in folk medicine for the treatment of several types of rheumatisms and diarrhoea. The focus of this work was to evaluate the *in vitro* antiproliferative and antioxidant activity and the *in vivo* hypoglycaemic and anti-inflammatory activity of *A. dioica* and identify the principal constituents of this plant.

**Methods:**

The crude methanol extract (EAD) and hexane (HF), chloroform (CF), ethyl acetate (EAF) and hydromethanol fractions (HMF) were evaluated for free radical scavenging activity using the DPPH assay. The EAD and EAF were assayed for hypoglycaemic activity in rats. The EAD was tested in an antiproliferation assay and for anti-inflammatory effects in paw oedema, in addition to myeloperoxidase activity induced by carrageenan (Cg) in mice. The EAF was assayed using chromatographic methods.

**Results:**

The fractionation of the EAF through chromatographic methods identified derivatives of the flavonoids quercetin and kaempferol. Among all the tested fractions, the ethyl acetate and hydromethanol fractions were the most potent, exhibiting an IC_50_ of 8.53 and 10.57 μg/mL, respectively, which is comparable to that of the commercial antioxidant butylated hydroxytoluene (BHT). The oral administration of the EAD (100 mg/kg) and EAF (15 mg/kg) inhibited the increase of glucose levels, resulting in a hypoglycaemic effect. The EAD (30 to 300 mg/kg) exhibited an anti-oedematogenic effect in Cg-induced paw oedema in a time- and dose-dependent manner. The results showed a reduction of MPO activity by *A. dioica* 6 h after the induction of paw oedema at all doses tested with maximal inhibition at 300 mg/kg.

**Conclusions:**

Our results reveal for the first time that compounds contained in the *A. dioica* leaves exert anti-inflammatory, hypoglycaemic, antiproliferative, and antioxidant effects. The antioxidant activity may be associated with the presence of flavonoids.

## Background

Plants have been used as a source of new medicinal compounds throughout history and continue to serve as the basis for many of the pharmaceuticals used today [1]. Standard experimental scientific methods are useful for the validation of ethnopharmacological knowledge regarding herbal medicine.

*Annona dioica* St. Hill. (Annonaceae) is a shrub found in Brazil throughout the states of São Paulo, Minas Gerais, Paraná and Mato Grosso. It is commonly referred to as “ceraticum”, “arixicum” and “ariticum”. In Brazil, the fruits and leaves of this plant are used to treat rheumatism, and the seeds are used to treat diarrhoea [2]. In Paraguay, the leaves are used to make tea or are gargled as an anti-catarrhal, while the edible fruits possess sedative properties. The seeds are used as insecticides or in the treatment of parasitic infections of the skin [3]. Chemical studies of the wood of *A. dioica* resulted in the isolation and characterisation of six alkaloids [4], while analysis of the leaves identified four flavonoids [5]. The cytotoxic activities of the flavonoids 3-*O*-[3^′^,6^′^-di-*Op*-hydroxycinnamoyl]-β-galactopyranosyl-kaempferol, 6^′^*Op*-hydroxycinnamoyl-β-galactopyranosyl-kaempferol and the flavonoid rich fraction of *A. dioica* were assayed *in vitro* against murine Ehrlich carcinoma and were shown to have significant antiproliferative effects [5]. The extracts of *A. dioica* Stem bark, heartwood subterranean, stem bark subterranean and of leaves were assayed *in vitro* against HCT-8 (human colon carcinoma), SF-295 (glioblastoma) and MDA-MB-435 (melanome) tumor cell presented high cytotoxic activity, over 75% [6]. The data indicate the importance of the continuity of phytochemical studies and especially in the evaluation against several human cancer cell lines, in addition other biological activities.

Flavonoids are naturally occurring phenolic compounds that are found in plants and are commonly consumed in the human diet [7]. Some flavonoids have been shown to display a number of interesting biological activities, such as antioxidant [8,9], antiviral, antifungal [10], anticancerous [11,12], anti-angiogenic [13] and anti-inflammatory activities [14,15].

The traditional use of medicinal plants is common in Brazil as an alternative to primary health care. Several plants, including Annona species, are used in Brazilian folk medicine. However, these plants are typically used without considering the toxicity and pharmacological aspects. Thus, it is essential to confirm the activity of this plant, especially the anti-inflammatory, hypoglycaemic and antiproliferative effects, in addition to identifying the specific mechanisms of action. In the present study, we evaluated the free radical scavenging, antiproliferative, hypoglycaemic and anti-inflammatory activity of the crude methanol extract (EAD) and fractions of *A. dioica* and identified the flavonoids found in the leaves of this plant.

## Methods

### Plant material

The leaves of *A. dioica* were collected in May 2009 in Dourados, in the state of Mato Grosso do Sul, Brazil. A voucher specimen (DDMS 4598) was deposited at the Herbarium of the Federal University of Grande Dourados, Dourados, MS, Brazil.

### Extraction, fractionation and isolation procedure

The air-dried and powdered aerial parts of *A. dioica* (850 g) were successively extracted via maceration with methanol. The extract was filtered and concentrated in a vacuum to obtain dry, crude methanol extract (EAD) (24.5 g). A portion of this extract (17.8 g) was dissolved in MeOH:H_2_O (1:1) and partitioned with *n*-hexane, chloroform and ethyl acetate. Each extract was dried over anhydrous sodium sulphate and concentrated under reduced pressure at a temperature not exceeding 35°C. This resulted in the *n*-hexane (HF, 3.8 g), chloroform (CF, 1.6 g) ethyl acetate (EAF, 6.2 g), and hydromethanol (HMF, 4.6 g) fractions. The EAF (3.2 g) was fractionated on Sephadex LH-20 using H_2_O, H_2_O: MeOH (75:25, 50:50, 25:75), and MeOH as the solvents to give ten sub-fractions (AD-1 to AD-10). The purification of sub-fraction AD-3 (505.2 mg) on Sephadex LH-20 using H_2_O, H_2_O: MeOH (75:25, 50:50, 25:75), and MeOH as the solvent yielded quercetin-3-*O*-galactoside (hyperoside) (32.4 mg) and mixture of quercetin-3-*O*-galactoside and kaempferol 3-*O*-galactoside (42.6 mg).

### Identification of the isolated compounds

The isolated compounds were identified analysis of their NMR data. The NMR measurements were carried out on a Varian Mercury Plus BB spectrometer operating at 300 MHz for ^1^H and 75.5 MHz for ^13^C using CD_3_OD as the solvent and tetramethylsilane (TMS) as the internal standard.

The structures of quercetin 3-*Oβ*-galactoside and the mixture of quercetin 3-*Oβ*-galactoside and kaempferol-3-*Oβ*-galactoside were elucidated using 1D and 2D NMR spectral data and by comparing their ^1^H and ^13^C NMR data with data reported in the literature [16,17].

Quercetin 3-*O*-β-galactoside: ^1^H NMR δ_H_ (300 MHz, CD_3_OD): 6.20 (1H, d, *J* = 2.1Hz), 6.39 (1H, d, *J* = 2.1Hz,), 7.66 (1H, dd, *J* = 8.7 e 2.4Hz,), 6.80 (1H, d, *J* = 8.7Hz), 7.51 (1H, d, *J* = 2.4Hz,), 5.37 (1H, d, *J* = 7.8Hz,); ^13^C NMR δ_c_ (75,5 MHz, CD_3_OD): 177.7, 164.4, 161.5, 156.5, 156.3, 148.7, 145.1, 133.7, 122.2, 121.1, 116.2, 115.3, 102.0, 104.2, 98.9, 93.7, 76.1, 73.4, 71.4, 68.2, 60.4.

Kaempferol-3-*O*-β-galactoside: ^1^H NMR δ_H_ (300 MHz, CD_3_OD): 6.19 (1H, d, *J* = 2.1Hz), 6.42 (1H, d, *J* = 2.1Hz,), 8.06 (2H, d, *J* = 8.7Hz,), 6.85 (2H, d, *J* = 8.7Hz), 5.39 (1H, d, *J* = 7.8Hz). RMN ^13^C δ_c_ (75,5 MHz, CD_3_OD): 177.8, 164.4, 161.5, 160.2, 156.6, 156.5, 133.4, 131.3, 131,3, 121.3, 115.4, 115.3, 104.2, 101.9, 98.9, 93.9, 76.1, 73.4, 71.4, 68.2, 60.4.

### Determination of total phenol content

The total phenolic content in the EAD was determined using the Folin-Ciocalteu method [18]. Specifically, 100 μL of EAD in methanol (1 g/L) was mixed with 1.0 mL of distilled water and 0.5 mL of Folin-Ciocaleu^’^s (1:10 v/v) reagent. After 3 min, 1.5 mL of a saturated solution of Na_2_CO_3_ (2%) was added. After 30 min, the absorbance was measured at 765 nm using a spectrophotometer. The quantification was carried out using a standard curve of gallic acid prepared in 80% methanol, and the results are expressed in milligrams of gallic acid equivalent per gram of extract. The equation for the gallic acid curve was y = 6.8502x + 0.0148, with a correlation coefficient of R = 0.9946. The methanol solution was used as a blank. All of the assays were carried out in triplicate.

### Determination of total flavonoids

To determine the level of flavonoids, 500 μL of EAD was mixed with 1.50 mL of 95% ethanol, 0.10 mL of 10% aluminium chloride (AlCl_3_.6H_2_O), 0.10 mL of acetate sodium (NaC_2_H_3_O_2_.3H_2_O) (1 M) and 2.80 mL of distilled water. The tubes were kept at room temperature for 40 min. The optical density was measured at 415 nm using a spectrophotometer. The same procedure was used for the analysis of the blank [19]. To calculate the concentration of flavonoids, we prepared a calibration curve (2.5, 5.0, 10.0, 20.0, 25.0, 50.0, 100.0 and 125.0 μg) using quercetin as the standard. We then used these data to generate a linear regression model, and the line equation was obtained and used for the calculation of the experimental samples. The results are expressed in milligrams of quercetin equivalents per gram of extract. The equation of the quercetin curve was y = 12.341x + 0.009, with a correlation coefficient of R = 0.9977. All of the assays were carried out in triplicate.

### DPPH free radical scavenging assay

The free radical scavenging activities of the crude methanol extract, and the hexane, chloroform, ethyl acetate and hydromethanol fractions were determined using the 1,1-diphenyl-1-picrylhydrazyl free radical (DPPH) method [20]. Various concentrations of the samples were added to 2 mL of a methanol DPPH solution (0.1 mM) that was prepared daily. The mixture was shaken and left to stand at room temperature in the dark. After 30 min, the absorbance was measured at 517 nm against a blank containing all of the reagents except for the test samples. All of the assays were carried out in triplicate. BHT was used as the positive control. The IC_50_ (the concentration required for 50% inhibition of DPPH) was calculated using the graph of % I (inhibition percentage) versus the extract concentration in mg/mL. The percentage of DPPH inhibition (% I) was calculated using the following equation: % I = (A_0_ - A/A_0_) x 100, where A_0_ is the absorbance of DPPH (control), and A is the absorbance of the sample with DPPH.

### Animals

The experiments were conducted using 30 male *Wistar* rats (150–230 g) and 30 male *Swiss* mice (25–35 g) provided by the Universidade Federal da Grande Dourados (UFGD). The animals were maintained under a 12-h light–dark cycle, with controlled humidity (60–80%) and temperature (22 ± 1°C). The animals were acclimatised to the experimentation room for at least 2 h before testing and were used only once throughout the experiments. All experimental procedures were carried out in accordance with the guidelines of the U.S. National Institute of Health and were approved by the ethics committee on laboratory animal use of the Centro Universitário da Grande Dourados (UNIGRAN) (Nbr. 118/2010).

### The oral glucose tolerance test in non-diabetic rats

This experiment was designed to evaluate the hypoglycaemic potential of the EAD and EAF in normal rats using the method described by Al-Awadi et al. (1985) [21]. The rats were orally treated daily for 5 d with crude methanol extract (EAD, 100 mg/kg) or the ethyl acetate fraction (EAF, 15 mg/kg). The reference drug, metformin (MET, 300 mg/kg), was also administered orally to the rats once a day. A separate control group of animals were orally administered the vehicle (saline plus tween 80 0.5%).

The oral glucose tolerance test (OGTT) for non-diabetic rats was performed according to the standard method [22]. Briefly, all groups were selected for the OGT test after food deprivation for 16 h. The baseline (B) glucose level was measured using a glucometer (Accuchek® Performa) prior to glucose administration (2 g/kg body weight).

After measuring the glucose baseline, each group received the specified oral treatment. Serum glucose in a blood sample from the tail vein was measured using a glucometer at 60 min. The data are expressed as the means ± standard error of mean (SEM). Statistical comparisons were performed using a one-way ANOVA followed by the Student-Newman-Keuls test, and the differences were considered statistically significant when P < 0.05. All statistical calculations and graphs were prepared using GraphPad Prism version 5.0 for Windows (GraphPad Software, San Diego, CA, USA).

### Carrageenan-induced paw oedema in mice

Five groups of mice (n = 6), were orally treated (p.o.) with the EAD (30–300 mg/kg). A separate control group was orally administered the vehicle (saline plus tween 80 0.5%). Another group of mice was treated subcutaneously with the anti-inflammatory drug dexamethasone (1 mg/kg). After 1 h, the animals received a 50-μl subcutaneous (s.c.) injection of Cg (300 μg/paw) dissolved in sterile 0.9% saline in the right hindpaw. The contralateral paw was injected with saline and used as the control. The thickness of paw oedema was measured using a digital micrometer [23] 1 h before any treatment and at several time points (1, 2, and 4 h) after the injection of Cg. The results are expressed in μm, and the difference between the basal and post-injection values are quantified as oedema.

### Determination of myeloperoxidase (MPO) activity

To investigate whether oral treatment with EAD (30 and 300 mg/kg) or vehicle could affect the cellular migration induced by Cg, the myeloperoxidase activity was measured. Animals were euthanised 6 h after Cg injection, as described previously [24]. For MPO activity, the tissue was homogenised in 5% (w/v) of 80 mM phosphate buffer (pH 5.4) containing 0.5% hexadecyltrimethylammonium bromide. The homogenate was centrifuged at 3200 rpm and 4°C for 20 min. Aliquots (30 μl) of each supernatant were mixed with 100 μl of 80 mM phosphate buffer, 85 μl of 0.22 M phosphate buffer and 15 μl of 0.017% H_2_O_2_ on a 96-well plate. The reaction was triggered with 20 μl of 3,3,3-tetramethylbenzidine (dissolved in N,N-dimethylformamide). The plate was kept at 37°C for 3 min, after which the reaction was stopped by adding 30 μl of 1.46 M sodium acetate, pH 3.0. The enzymatic activity was determined by measuring the optical density at 630 nm and is expressed as the mOD per milligram of protein.

### Antiproliferative assay

The National Cancer Institute, Frederick MA/USA, kindly provided nine human cancer cell lines: U251 (glioma, CNS), UACC-62 (melanoma), MCF-7 (breast), NCI-ADR/RES (ovarian expressing phenotype multiple drug resistance), 786–0 (renal), NCI-H460 (lung, non-small cells), PC-3 (prostate), OVCAR-03 (ovarian), k-562 (leukaemia) and HT29 (colon). VERO (green monkey kidney cells), a normal cell line, was also used. The stock and experimental cultures were grown in medium containing 5 mL of RPMI 1640 (GIBCO BRL) supplemented with 5% foetal bovine serum (GIBCO BRL).

The stock cultures were grown in 5 mL of RPMI-1640 (GIBCO BRL) supplemented with 5% foetal bovine serum (FBS, GIBCO). A penicillin:streptomycin mixture (1000 U/mL:1000 μg/mL, 1 mL/L RPMI, Nutricel) was added to the experimental cultures.

The cells were plated in 96-well plates (100 μL cells/well) and exposed to different concentrations of the EAD (0.25, 2.5, 25 and 250 μg/mL) in DMSO/RPMI (0.1% v/v) at 37°C and 5% CO_2_ for 48 h. The final DMSO concentration did not affect cell viability. The cells were then fixed with a trichloroacetic acid solution (50%, v/v), and cell proliferation was determined via spectrophotometric quantification (540 nm, Molecular Devices Versa Max Microplate Reader) of the cellular protein content using a sulphorhodamine B assay [25]. Doxorubicin (0.025-25 μg/mL) was used as a positive control. Three measurements were obtained: first at time zero (*T*_*o*_, at the beginning of incubation) and then 48 h post-incubation for both the compound-free (*C*) and tested (*T*) cells. Cell proliferation was determined using the equation 100 x [(*T* - *T*_*o*_)/C - *T*_*o*_. A cytostatic effect was observed when *T* ≥ *T*_*o*_*,* while a cytocidal effect occurred when *T* < *T*_*o*_. The experiments were performed in triplicate.

### Chemicals

1,1-Diphenyl-2-picrylhydrazyl (DPPH), butylated hydroxytoluene (BHT) λ-carrageenan (Cg), Tween 80, dexamethasone, trichloroacetic acid and doxorubicin were purchased from Sigma Chemical Co. (MO, USA). Analytical-grade methanol, hexane, anhydrous sodium sulphate and DMSO were obtained from Vetec (RJ, Brazil).

## Results and discussion

The free radical scavenging activity was initially evaluated for the crude methanol extract of *A. dioica* (EAD). The results (Table 1) show that the EAD possesses significant free radical scavenging activity, with an IC_50_ of 17.84 μg/mL. The fractionation of this extract by solvent partition yielded the hexane (HF), chloroform (CF), ethyl acetate (EAF), and hydromethanol (HMF) fractions, which were tested for their antioxidant ability toward the radical DPPH. A comparison of the obtained IC_50_ data (Table 1) indicated potent activity for the EAF and HMF fractions, with IC_50_ values of 8.53 μg/mL and 10.53 μg/mL, respectively, which was comparable to that of the commercial antioxidant BHT.

**Table 1:** **DPPH free radical scavenging activity (IC**_**50**_**) for EAD, HF, CF, EAF, and HMF fractions from *A. dioica***

**Sample**	**IC**_**50**_**μg/mL**
EAD	17.84
HF	101.66
CF	98.35
EAF	8.53
HMF	10.57
BHT^a^	16.8

^a^BHT (butylated hydroxytoluene) was used as positive control; EAD (crude methanol extract); HF (hexane fraction); CF (chloroform fraction); EAF (ethyl acetate fraction); HMF (hydromethanol fraction).

After determination of the antioxidant potential, we determined the levels of total phenols and flavonoids in the crude methanol extract. The EAD had high levels of total phenols and flavonoids with values of 187.77 mg/g and 733.20 mg/g, respectively. The total polyphenol and radical scavenging activity have been determined in some fruits of the genus *Annona,* namely *A. muricata*[26]*, A. cherimolia*[27] and *A. squamosa*[28].

Flavonoids are very important for the treatment of many diseases due to their potent antioxidant nature. Some authors have reported that flavonoids such as rutin (quercetin-3-rutinoside) and quercetin show antioxidant activity [14,15]. The antioxidant or free radical scavenging activity of flavonoids has been related to the number and position of free hydroxyl groups, which could be a result of their hydrogen donating ability [29,30]. The antioxidant activity and radical-scavenging activity of flavonoids depend highly on their structure, especially the presence of a free C-3–OH, a free C-4–OH, a double bond between C-2 and C-3, and an O-dihydroxy group in the B-ring [31]. The isolated flavonoids were a good match for these requirements; the only exception is the absence of a free C-3–OH. The antioxidant effects observed for EAD, EAF and HMF are most likely due the presence of quercetin and/or kaempferol and phenolic compounds based on the DPPH free radical activity of these flavonoids reported in the literature [32].

The *in vitro* antiproliferative assay of the EAD was evaluated against VERO and nine human cancer cell lines. Three response parameters (GI_50_, TGI, and LC_50_) were calculated for the cell lines tested, and the results are summarised in Table 2. The GI_50_ value (growth inhibitory activity) refers to the drug concentration that produces a 50% reduction in cellular growth when compared to the untreated control cells. The TGI (cytostatic activity) concentration that produces 0% cell growth or cytostatic effect and the LC_50_ (cytotoxic activity) refer to the drug concentrations necessary for total growth inhibition and for killing 50% of the cells, respectively.

**Table 2:** **GI**_**50**_**, TGI and LC**_**50 **_**(μg/mL) for EAD and Dox**

	**EAD**			**Dox**		
***Cancer cell lines***	**GI**_**50**_	**TGI**	**LC**_**50**_	**GI**_**50**_	**TGI**	**LC**_**50**_
**U251 (glioma)**	83.47			5.07	23.45	
**UACC-62 (melanoma)**	> 100			0.06	0.86	
**MCF7 (breast)**	11.10	> 100		0.14	24.52	
**NCI/ADR-RES (ovarian resistance)**	1.95			1.74	23.92	
**786-0 (renal)**	> 100			0.22	1.51	
**NCI-H460 (lung)**	0.03	> 100		0.05		
**OVCAR-3 (ovarian)**	6.38			0.30	1.72	
**HT29 (colon)**	0.10	4.10	> 100	2.12	7.31	20.86
**K-562 (leukemia)**	31.13			0.90		
**Vero**				1.40	11.36	

EAD (crude methanol extract); dox (doxorubicin); GI_50_ (growth inhibitory activity); TGI (cytostatic activity); LC_50_ (cytotoxic activity); IC_50_ >100 (μg/mL) was considered not active.

The results demonstrate that the EAD possesses anticancer activity with GI_50_ values in the range of 0.03 – 83.47 μg/mL. Regarding the sensitivity against particular cell lines, the EAD was particularly effective in NCI-H460 lung cells (GI_50_ 0.03 μg/mL), HT29 colon cells (GI_50_ 0.10 μg/mL and TGI 4.10 μg/mL), NCI/ADR-RES ovarian expressing phenotype multiple drug resistance cells (GI_50_ 1.95 μg/mL), OVCAR-3 ovarian cells (GI_50_ 6.38 μg/mL) and MCF7 breast cells (GI_50_ 11.10 μg/mL). Lung cancer is among the most common types of cancer throughout the world and shows only a modest response to the chemotherapeutic treatments available. For this type of cancer, monotherapy presents only a partial response in 15% to 20% of cases, and even with combined treatments, the therapeutic effect does not exceed 40% - 50%.

The criteria of the American National Cancer Institute to consider a crude extract promising for further purification is a GI_50_ lower than 30 μg/mL [33]. A number of studies have demonstrated anticancer activity associated with flavonoids [34].

Extracts from different species of the Annonaceae family have been shown to be active. The strongest cytotoxic activities were detected for the ethanol extracts of the *A. crassiflora* root bark [35].

**Figure 1:**
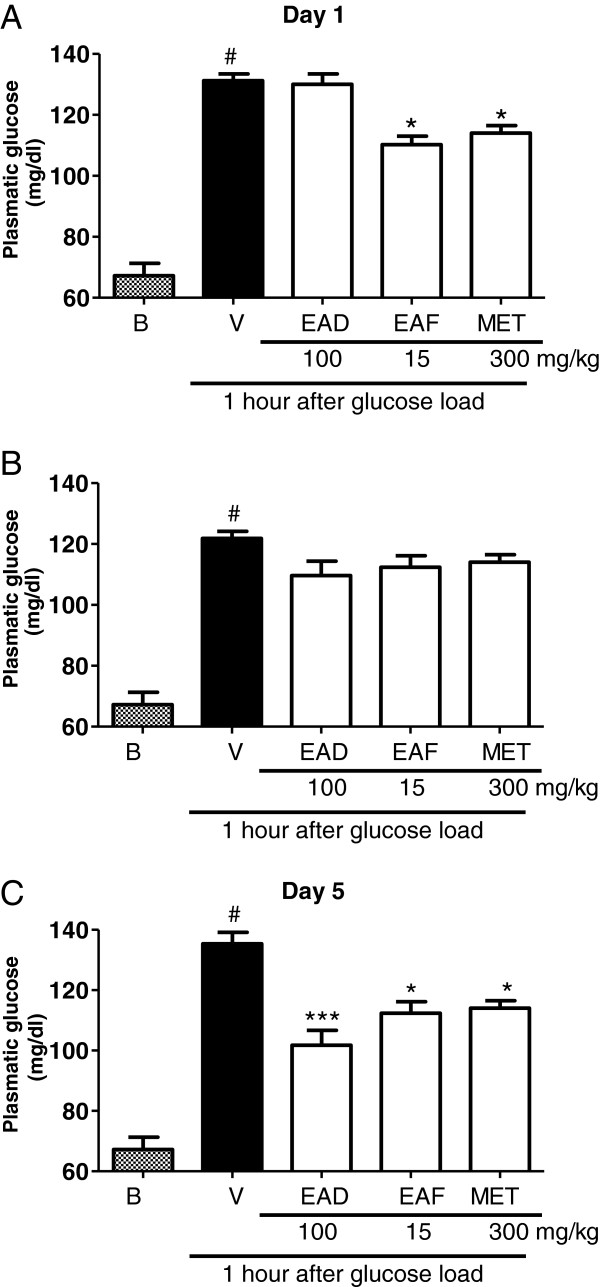
**Effects of crude methanol extract (EAD), ethyl acetate fraction (EAF) on increasing in glucose plasmatic levels in normoglicemic rats.** In **A**, **B**, and **C** the results of daily treatment in day 1, 3 and 5 with vehicle (control group), EAD (100 mg/kg), EAF (15 mg/kg), and metformin (MET, 300 mg/kg) in nomal rats. Results are expresses as mg/dL compared with vehicle (V) vs treated group. *P < 0.05, ***P < 0,0001, one-way ANOVA followed by Student-Newman-Keuls.

On the first day of oral treatment, EAF (15 mg/kg) as well as MET (300 mg/kg), but not EAD (Figure 1A) and quercetin 3-*β-*galactoside (results not shown), inhibited the increase in glucose levels with inhibitions of 16 ± 2% and 13 ± 2%, respectively (Figure 1A) (results not shown). On the fifth day, EAD (100 mg/kg), EAF (15 mg/kg), quercetin 3-*β-*galactoside (1.5 mg/kg; results not shown) and MET (300 mg/kg) significantly reduced the glucose increase, with inhibitions of 22 ± 4%, 14 ± 3%, 16 ± 3% and 13 ± 2%, respectively (Figure 1C). Together, these results show that the extract from the leaves of *A. dioica* exhibited hypoglycaemic effects and that the flavonoid derivatives quercetin and kaempferol present in EAD and EAF are responsible, at least in part, for the observed hypoglycaemic activity in non-diabetic rats. We cannot confirm that the flavonoids are the only compounds present in the extract responsible for this action because the EAD showed efficacy in relation to all groups, including the metformim group.

**Figure 2:**
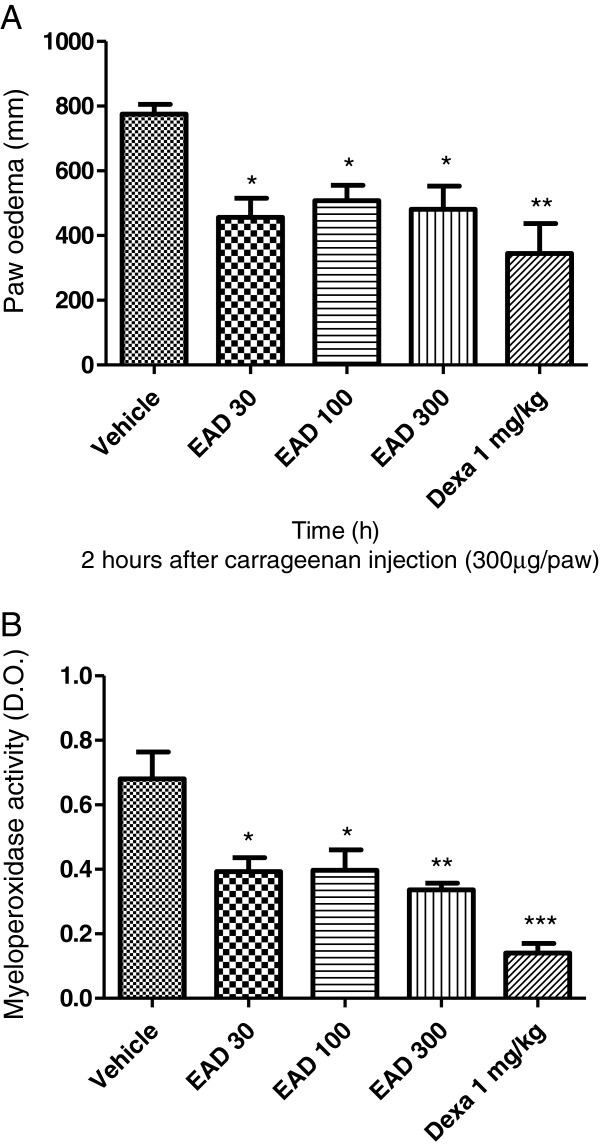
**Effects of crude methanol extract (EAD) on oedema formation and myeloperoxidase activity induced by carrageenan in mice.** In **A**, the results of single oral treatment with vehicle (control group), EAD (30, 100 and 300 mg/kg), and dexamethasone (Dexa, 1 mg/kg, s.c.) in oedema formation at 2 hs time point (**A**) and in myeloperoxidase activity at 6 hs after (**B**) carrageenan (300 mg/kg) in mice. Results are expresses as mg/dL compared with vehicle (V) vs treated group. *P < 0.05, **P < 0,01, ***P < 0,0001, one-way ANOVA followed by Student-Newman-Keuls.

The potential anti-inflammatory or anti-oedema properties of pharmacological substances in experimentally induced rodent models are assessed using the digital water plethysmometer or micrometer methodology. The micrometer method was used to measure acute paw oedema in order to screen for anti-inflammatory activity [23]. The injection of Cg into the paw induced an oedema that peaked at 2 h (Figure 2A). Oral treatment with EAD (30, 100 and 300 mg/kg) significantly inhibited oedema formation. The inhibitions for each dose were 39 ± 8% (30 mg/kg), 32 ± 6% (100 mg/kg), and 36 ± 10% (300 mg/kg) (Figure 2A). In addition, the inhibition observed in the dexamethasone-treated group was also significant (Figure 2A). These results suggest that EAD contains compounds that exert anti-oedematogenic properties.

The marked increase in the myeloperoxidase activity (indirect evidence for neutrophil influx) into the paw indicated an inflammatory process induced by Cg, instead of oedema formation only. The injection of Cg (300 μg/paw) increased MPO activity after 6 h, and oral treatment with EAD (30, 100 and 300 mg/kg) inhibited the increase in MPO activity induced by Cg. The observed inhibitions were 42 ± 6% (30 mg/kg), 42 ± 9% (100 mg/kg), and 51 ± 3% (300 mg/kg). The positive control (dexamethasone) was also able to inhibit MPO activity compared to the control group (Figure 2B). This result corroborates the anti-oedematogenic properties of EAD (Figure 2A) and shows the anti-inflammatory efficacy of EAD, suggesting that these effects are comparable with those of commercially available anti-inflammatory steroidal products, such as dexamethasone.

## Conclusions

To the best of our knowledge, this is the first systematic screening for phenols and flavonoids and the first evaluation of the biological activities of the plant *A. dioica*. Our study demonstrates the potential anti-oxidant, anticancer, hypoglycaemic and anti-inflammatory properties of *Annona dioica*. This effect can be attributed the presence of flavonoids.

## Abbreviations

BHT: butylated hydroxytoluene; Cg: Carrageenan; CF: Chloroform fraction; CD_3_OD: Methanol-d_4_; DPPH: 1,1-diphenil-1-picrylhydrazyl; EAD: Crude methanol extract; EAF: Ethyl acetate fraction; FBS: Fetal bovine serum; GI_50_: Growth inhibitory; HF: Hexane fraction; HMF: Hydromethanol fraction; HT-29: Colon; IC_50_: Concentration inhibitory; %I: Inhibition percentage; k-562: Leukemia; LC_50_: Cytotoxic activity; MET: Metformin; MPO: Myeloperoxidase; MCF-7: Breast; MHz: MegaHertz; NCI-ADR/RES: Ovarian expressing phenotype multiple drug resistence; NCI-H460: Lung; NMR: Nuclear magnetic resonance; OGTT: Oral glucose tolerance test; OVCAR-03: Ovarian; PC-3: Prostate; TGI: Cytostatic activity; TMS: Tetramethylsilane; U251: Glioma; UACC-62: Melanoma; VERO: Green monkey Kidney; 786–0: Renal.

## Competing interests

The authors declare that they have no competing interests.

## Authors’ contributions

ASNF and CRFV designed the study, performed the extraction/fractionation of the extract, performed the isolation/structure elucidation of the compounds, determined the levels of total phenols and flavonoids, assessed the antioxidant activity and helped in manuscript writing and editing. CALK, FFN and EKKI participated in the hypoglycaemic and anti-inflammatory assays. MAF assisted in the antiproliferative assay. MCV participated in the collection of plant material. All authors read and approved the final manuscript.

## Pre-publication history

The pre-publication history for this paper can be accessed here:

http://www.biomedcentral.com/1472-6882/13/14/prepub
